# Identification of WNT16 as a Predictable Biomarker for Accelerated Osteogenic Differentiation of Tonsil-Derived Mesenchymal Stem Cells *In Vitro*

**DOI:** 10.1155/2019/8503148

**Published:** 2019-09-10

**Authors:** Yu-Hee Kim, Kyung-Ah Cho, Hyun-Ji Lee, Minhwa Park, Han Su Kim, Joo-Won Park, So-Youn Woo, Kyung-Ha Ryu

**Affiliations:** ^1^Department of Microbiology, College of Medicine, Ewha Womans University, Seoul 07804, Republic of Korea; ^2^Department of Otolaryngology, College of Medicine, Ewha Womans University, Seoul 07985, Republic of Korea; ^3^Department of Biochemistry, College of Medicine, Ewha Womans University, Seoul 07804, Republic of Korea; ^4^Department of Pediatrics, College of Medicine, Ewha Womans University, Seoul 07804, Republic of Korea

## Abstract

The application of mesenchymal stem cells (MSCs) for treating bone-related diseases shows promising outcomes in preclinical studies. However, cells that are isolated and defined as MSCs comprise a heterogeneous population of progenitors. This heterogeneity can produce variations in the performance of MSCs, especially in applications that require differentiation potential *in vivo*, such as the treatment of osteoporosis. Here, we aimed to identify genetic markers in tonsil-derived MSCs (T-MSCs) that can predict osteogenic potential. Using a single-cell cloning method, we isolated and established several lines of nondifferentiating (ND) or osteoblast-prone (OP) clones. Next, we performed transcriptome sequencing of three ND and three OP clones that maintained the characteristics of MSCs and determined the top six genes that were upregulated in OP clones. Upregulation of WNT16 and DCLK1 expression was confirmed by real-time quantitative PCR, but only WNT16 expression was correlated with the osteogenic differentiation of T-MSCs from 10 different donors. Collectively, our findings suggest that WNT16 is a putative genetic marker that predicts the osteogenic potential of T-MSCs. Thus, examination of WNT16 expression as a selection criterion prior to the clinical application of MSCs may enhance the therapeutic efficacy of stem cell therapy for bone-related complications, including osteoporosis.

## 1. Introduction

Osteoporosis is an age-related bone disease that involves a gradual loss of bone mass and an associated increase in fracture risk. Osteoporotic fracture not only limits patients' activities of daily living but also increases their morbidity and mortality. Osteoporotic fracture is the second most burdensome disease considering years of life lost, with an estimated 5.8 million years lost due to the disease worldwide in 2000 [1]. Given that osteoporosis is a disease that can affect all human beings and that becomes more prevalent with extensions in life expectancy, effective therapeutics that prevent or delay the progression of osteoporosis are in high demand. Current treatment options for osteoporosis include drugs that inhibit bone resorption or stimulate bone generation [2]. However, these drugs have limited therapeutic efficacies and can cause serious side effects under long-term treatment. Therefore, alternative therapeutic strategies, such as cell-based therapy, should be considered.

Mesenchymal stem cells (MSCs) are characterized by self-renewal, multipotency, and immunomodulatory properties. Their capacity of multilineage differentiation, especially into osteoblastic cells, has been employed in stem cell-based therapy for bone defects due to genetic disorders or injuries [3, 4]. As MSCs are a heterogeneous population of progenitor cells, their osteogenic capacity varies between donors and within cell populations [5, 6]. Attempts have been made to identify biomarkers associated with the osteoblastic lineage commitment of MSCs to enable the selection of more therapeutically efficacious MSCs and the prediction of therapeutic outcomes. Surface antigen screening of human bone marrow-derived MSCs (BM-MSCs) shows that CD106-expressing cells readily differentiate into adipocytes but not osteoblasts [6]. Other studies describe markers that can distinguish differentiation potentials using proteomic or transcriptomic approaches [7–9]. Thus, a deeper understanding of MSC heterogeneity and the properties of clonally expanded populations could enable the selection of specific subpopulations for precise clinical uses.

The human tonsil is a tissue source for MSCs, termed tonsil-derived MSCs (T-MSCs). Previously, we and others isolated and characterized MSCs from human palatine tonsils [10, 11] and confirmed that these cells proliferate rapidly [12, 13] and do not exhaust their self-renewal properties, mesodermal differentiation potential, and expression of embryonic stem cell markers until passage 15 [14]. T-MSCs retain their osteogenic differentiation capacity in long-term culture [14] and possess bone regenerative properties as demonstrated in senile and postmenopausal osteoporosis mouse models [15, 16]. The therapeutic efficacy of MSCs can be enhanced by embedding MSCs in gelatin hydrogel or direct intratibial injection of T-MSCs [17]. MSCs promote bone regeneration through self-differentiation into osteoblasts and the exertion of paracrine effects, including enhanced osteoblast differentiation and the regulation of osteoclast activity [15, 18]. Although recent studies of T-MSCs show promising outcomes, heterogeneity in the T-MSC population could produce variations in therapeutic efficacy and thus be a major obstacle to their clinical use.

In this study, we employed a single-cell cloning method to characterize monoclonal cells isolated from T-MSCs with high osteogenic potential. The expression of MSC surface markers, doubling time, and osteogenic differentiation were assessed in monoclonal cells to select nondifferentiating (ND) or osteoblast-prone (OP) clones. Transcriptome sequencing was performed to identify genes differentially expressed in OP clones, and their expressions were examined in several lines of T-MSCs isolated from different donors. Furthermore, we correlated selected gene expression with osteoblast differentiation to predict the efficacy of cell-based therapy.

## 2. Materials and Methods

### 2.1. Cell Culture

Tonsils were obtained from patients undergoing tonsillectomy at the Department of Otorhinolaryngology, Head and Neck Surgery at Ewha Womans University Mok-Dong Hospital (Seoul, South Korea; approved by the Institutional Review Board; no. ECT2011-09-003), and isolation of MSCs was performed as previously reported [11]. BM-MSCs were purchased from ATCC (Manassas, VA) and Severance Hospital Cell Therapy Center (Seodaemun-gu, Seoul, Korea). Adipose-derived MSCs (AD-MSCs) and Wharton's jelly-derived MSCs (WJ-MSCs) were purchased from PromoCell (Heidelberg, Germany). Cells were cultured in DMEM low-glucose medium (Welgene, Gyeongsan, Korea) containing 10% fetal bovine serum (FBS), 100 IU/ml penicillin, and 100 *μ*g/ml streptomycin (Welgene). The culture medium was changed every 3–4 days and maintained at 37°C in a humidified atmosphere of 5% CO_2_.

### 2.2. Mesodermal Differentiation

To induce mesodermal differentiation, confluent T-MSCs were incubated using a StemPro osteogenesis or adipogenesis differentiation kit (Thermo Fisher Scientific, Waltham, MA) for 3 weeks. Osteoblast differentiation was determined by fixing cells with 60% isopropanol and staining with Alizarin red S solution (Sigma-Aldrich, St. Louis, MO) for 3 min. After washing cells with distilled water four times, matrix mineralization was quantified by eluting the stain with 10% cetylpyridinium chloride (Sigma-Aldrich) and measuring absorbance at 570 nm. Adipocyte differentiation was examined by fixing cells in 4% paraformaldehyde followed by staining with oil red O solution (Sigma-Aldrich). To induce chondrocyte differentiation, 2 × 10^6^ cells were pelleted in a conical tube by centrifugation at 1,300 rpm for 5 min at room temperature and incubated with a StemPro chondrogenesis differentiation kit (Thermo Fisher Scientific) for 3 weeks. Paraffin-embedded sections (5 *μ*m thickness) were prepared and stained with Alcian blue solution (Sigma-Aldrich) for 30 min followed by counterstaining with nuclear fast red solution (Sigma-Aldrich) for 5 min. Cell staining was observed using phase-contrast microscopy.

### 2.3. Single-Cell Cloning

Cloning by limiting dilution was performed by manual dilutions, plating the selected parental T-MSCs at one cell per well of a 96-well plate. After 2–3 weeks, clones rising from single cells were collected and transferred to a 24-well plate. After 4–8 days and before reaching confluency, cells were passaged to a 100 mm dish and cultured for 1 additional week for further expansion of monoclonal cells. Cells were induced for osteogenic differentiation and categorized into ND or OP clones according to their osteogenic capacities. ND or OP clones were then frozen in cell freezing medium containing 10% DMSO (Mylan, Canonsburg, PA) and stored in liquid nitrogen.

### 2.4. Flow Cytometry

MSC surface markers were examined by flow cytometry analysis. Parental T-MSCs or clones were resuspended in PBS-based buffer containing 0.5% FBS and 0.1% (*w*/*v*) sodium azide and incubated with the following antibodies for 30 min on ice: fluorescein isothiocyanate-labeled anti-human CD11b (ICRF44, mouse IgG_1_; BioLegend, San Diego, CA), Alexa Fluor 488 anti-human CD34 (561, mouse IgG_2a_; BioLegend), peridinin chlorophyll protein-labeled anti-human CD45 (2D1, mouse IgG_1_; BioLegend), allophycocyanin-labeled anti-human CD73 (AD2, mouse IgG_1_; BioLegend), phycoerythrin-labeled anti-human CD90 (5E10, mouse IgG_1_; BD Biosciences, San Jose, CA), and phycoerythrin-labeled anti-human CD105 (43A3, mouse IgG_1_; BioLegend). Marker expression was measured using a NovoCyte flow cytometer and analyzed using NovoExpress software (ACEA Biosciences, San Diego, CA).

### 2.5. Cell Proliferation Assay

To determine the doubling time of clones, 1 × 10^5^ cells were seeded into 60 mm culture dishes. After 96 h of incubation, cells were harvested, and live cells were counted using a hemocytometer. The doubling time of monoclonal cells was calculated using the Patterson formula: doubling time (h) = [{(*T*–*T*_0_)(log_2_)}/(log *N*–log *N*_0_)], where *T* is the time (h) and *N* is the number of cells.

### 2.6. Transcriptome Sequencing

RNA was extracted from three ND and three OP clones using a NucleoSpin RNA kit (Macherey-Nagel, Düren, Germany) according to the manufacturer's instructions. DNA contamination was assessed using a PicoGreen dsDNA assay kit (Thermo Fisher Scientific), and RNA quantity and quality were examined using an Agilent 2100 Bioanalyzer (Agilent Technologies, Santa Clara, CA) with an RNA integrity number ≥ 7. A cDNA library was generated using a TruSeq Stranded mRNA sample prep kit (Illumina, San Diego, CA), and transcriptome sequencing was performed using a TruSeq 3000/4000 SBS kit and HiSeq 4000 sequencer (Illumina) with 101 bp paired-end reads per sample (Macrogen, Seoul, Korea).

### 2.7. Differentially Expressed Gene (DEG) Analysis

Sequenced cDNA fragments were mapped to the human genomic DNA reference (USCS hg19) using HISAT2 [19]. StringTie was used for transcript assembly and fragments per kilobase of transcript per million mapped reads (FPKM) determination [20]. FPKM value was used for assessing the relative expression of a transcript. For DEG analysis, genes with a FPKM value of 0 in every sample were excluded (7,985 out of 27,685 genes), values of log_2_ (FPKM + 1) were calculated, and quantile normalization was performed using the preprocessCore R library. Transcripts with fold change > 1.5 and independent *t*-test raw *p* value < 0.05 were selected as DEGs. Hierarchical clustering of DEGs was performed using Euclidean distance and complete linkage, and gene set enrichment analysis of DEGs was conducted based on gene ontology (GO; http://geneontology.org/).

### 2.8. Real-Time Quantitative Polymerase Chain Reaction (PCR)

Reverse transcription was performed using 1 *μ*g RNA and 5× Reverse Transcription Master Premix (ELPIS-Biotech, Daejeon, Korea) according to the manufacturer's instructions. Quantitation of target gene expression was performed by mixing cDNA with a primer pair (0.4 *μ*M) and using a SensiFAST SYBR Hi-ROX kit (Bioline, London, UK). Gene amplification was conducted using 40 cycles of a 15 s denaturation step at 95°C and 1 min amplification and signal acquisition step at 62°C using a StepOnePlus Real-Time PCR System (Applied Biosystems, Foster City, CA). The relative expression levels of target genes were determined using GAPDH as a housekeeping gene according to the following calculation: 2^(GAPDH Ct–target gene Ct)^. Primer sequences are listed in Table 1.

### 2.9. Statistical Analysis

Statistical analyses were performed using Student's *t*-tests or one-way ANOVA in conjunction with Tukey's post hoc tests using GraphPad Prism 8 (GraphPad Software, La Jolla, CA). Pearson's correlation coefficient was calculated to analyze correlation between degree of mineralization and gene expression. Data are presented as mean ± standard error of the mean (SEM), and a *p* value < 0.05 was considered to indicate statistical significance.

## 3. Results

### 3.1. Generation of Monoclonal T-MSC Subpopulations

**Figure 1:**
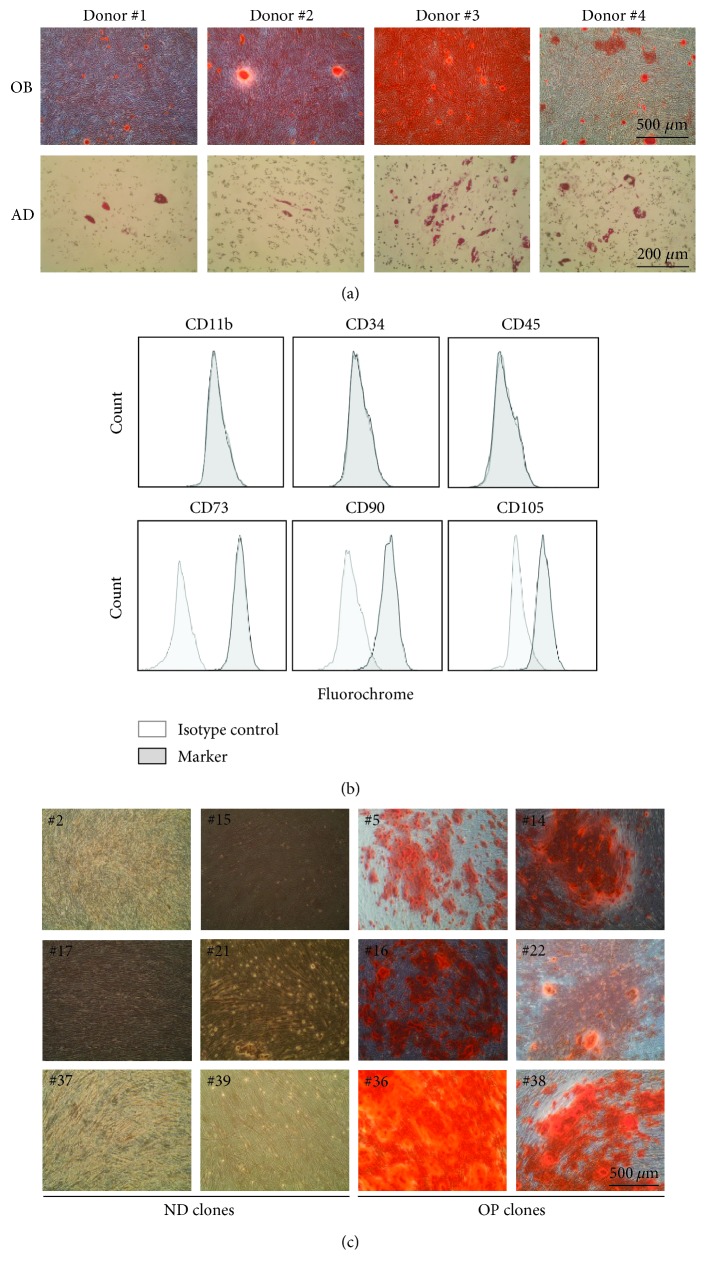
Generation of monoclonal T-MSC subpopulations. (a) Osteogenic (OB) and adipogenic (AD) differentiation was induced in T-MSCs from four different donors. Matrix mineralization and lipid droplet formation were examined by Alizarin red S and oil red O staining, respectively, under phase-contrast microscopy (100x magnification for OB and 200x magnification for AD). (b) Surface marker expression in T-MSCs selected for further single-cell cloning was examined by flow cytometry. (c) OB differentiation was induced in monoclonal cells, and Alizarin red S staining was used to assess matrix mineralization. Representative images of six nondifferentiating (ND) and six osteoblast-prone (OP) clones were shown (100x magnification).

To select a donor cell line with superior osteogenic potential, we induced osteogenic or adipogenic differentiation of primary T-MSCs isolated from four donors. Osteoblast differentiation was examined by Alizarin red S staining and adipocyte differentiation by oil red O staining. T-MSCs from all donors successfully underwent osteogenic and adipogenic differentiation, but each showed a different degree of differentiation potential (Figure 1(a)). Of these cells, we selected donor #2 to produce monoclonal cell colonies because these cells showed the highest differentiation potential toward osteoblasts and the lowest toward adipocytes. Flow cytometry analysis to assess the expression of MSC markers showed that donor #2 parental cells expressed CD73, CD90, and CD105 but not CD11b, CD34, or CD45 (Figure 1(b)). Next, we performed single-cell cloning through limiting dilution. Cells were first seeded in a 96-well plate. Clonally expanded cells were transferred to a 24-well plate and further proliferated in a 100 mm culture dish. We obtained 62 clonally derived T-MSC subpopulations through performing this limiting dilution method twice. Differentiation toward osteoblasts was induced for 3 weeks, and matrix mineralization was determined by Alizarin red S staining. Out of the 62 clones, 11 were successfully differentiated into osteoblasts. We selected six OP (clone #5, #14, #16, #22, #36, and #38) and six ND (clone #2, #15, #17, #21, #37, and #39) clones (Figure 1(c)). The selected ND clones showed similar levels of proliferating capacity to OP clones.

### 3.2. Selection of Clones for Transcriptome Sequencing

**Figure 2:**
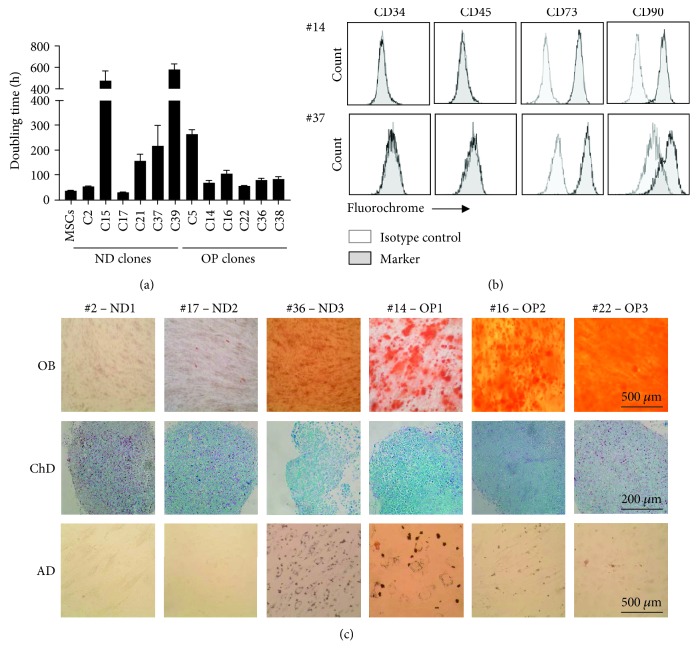
Selection of clones for transcriptome sequencing. Screening of MSC characteristics of 12 clones was performed. (a) Doubling time (h) of parental T-MSCs and clones; data are shown as mean ± SEM. (b) Surface marker expression in clones; representative images show changes in CD90 expression. (c) Clones were induced for OB, chondrogenic (ChD), and AD differentiation and stained with Alizarin red S, Alcian blue, and oil red O staining, respectively (100x magnification for OB and AD, 200x magnification for ChD). Representative images of three ND and three OP clones selected for further analyses were presented.

We next screened characteristics of the 12 selected clones. Assessment of doubling time showed that some clones maintained their self-renewal capacity, whereas others lost this capacity, possibly due to the cryopreservation and thawing procedures (Figure 2(a)). Examination of MSC surface marker expression showed a reduction in CD90 expression in some clones (Figure 2(b)). We also induced differentiation of clones into osteoblasts, chondrocytes, or adipocytes (Figure 2(c)). After excluding clones that showed a doubling time longer than 100 hours or low expression of CD90, three ND and three OP clones were selected for transcriptome sequencing based on their osteogenic potential. Clone #36 seemed to lose its osteogenic potential after cryopreservation and thawing and was thus classified as ND at the time of transcriptome sequencing.

### 3.3. Transcriptome Sequencing

**Figure 3:**
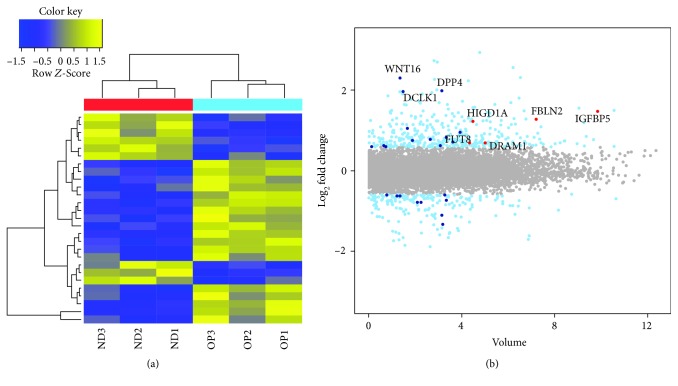
Transcriptome sequencing. Transcriptome sequencing of three ND and three OP clones was performed, followed by further analysis of DEGs. (a) Heat map of 27 DEGs showing a fold change > 1.5 and *p* value < 0.05 between ND and OP clones. (b) Volume plot showing the top five DEGs with high expression volume marked with red dots (IGFBP5, FBLN2, HIGD1A, DRAM1, and FUT8) and three other highly upregulated genes (WNT16, DPP4, and DCLK1).

During transcriptome sequencing, averages of 71 million 101-base long reads were processed, and approximately 98% of reads were mapped to the human genomic DNA reference. Mapped reads were assembled to the known transcripts and condensed into FPKM expression values. DEG analysis was performed between ND and OP clones using FPKM values, and results were visualized by a heat map of 27 genes with a log_2_ base fold change > 1.5 and raw *p* value < 0.05 (Figure 3(a)). As these clonally expanded cells originated from the same CD73-, CD90-, and CD105-positive parental cells, a small number of genes were differentially expressed between the two groups. A volume plot was generated to show the expression volume and fold change between ND and OP clones. The top five DEGs with high expression volume (insulin-like growth factor binding protein 5, IGFBP5; fibulin 2, FBLN2; hypoxia-inducible domain family member 1A, HIGD1A; DNA damage-regulated autophagy modulator 1, DRAM1; and fucosyltransferase 8, FUT8) are marked as red dots, and three other highly upregulated genes (Wnt family member 16, WNT16; dipeptidyl peptidase 4, DPP4; and doublecortin-like kinase 1, DCLK1) are indicated in Figure 3(b). Transcripts that were up- or downregulated in OP clones with a log_2_ base fold change > 2 and raw *p* value < 0.05 are listed in Table 2. The top six upregulated genes were selected for further analyses. Next, gene set enrichment of DEGs was analyzed according to the GO categories of biological process, molecular function, and cellular component (Figure 3(b)). GO functional analyses revealed that genes highly expressed in OP clones were located in intracellular as well as extracellular regions and were involved in the regulation of cellular and biological processes via protein binding, ion binding, and catalytic activity. Next, gene set enrichment of DEGs was analyzed according to the GO categories of biological process, molecular function, and cellular component (Supplementary [Supplementary-material supplementary-material-1]). GO functional analyses revealed that genes highly expressed in OP clones were located in intracellular as well as extracellular regions and were involved in the regulation of cellular and biological processes via protein binding, ion binding, and catalytic activity.

### 3.4. Expression of Markers in a Heterogeneous Population of Progenitors

**Figure 4:**
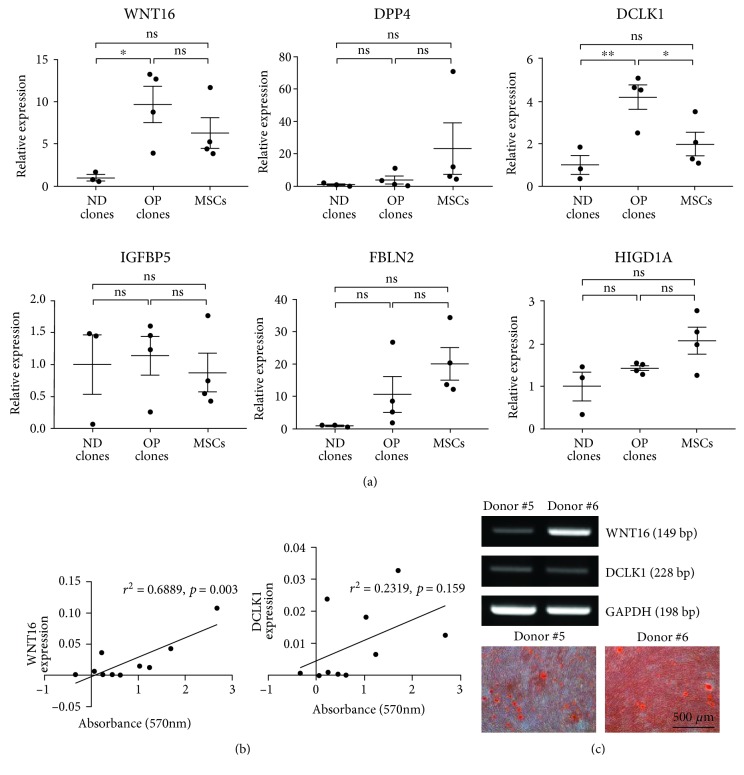
Expression of markers in a heterogeneous population of progenitors. (a) Expression of the six selected genes (WNT16, DPP4, DCLK1, IGFBP5, FBLN2, and HIGD1A) was confirmed in ND and OP clones as well as T-MSCs from four different donors using real-time quantitative PCR. Data are shown as mean ± SEM (ns: not significant, ^∗^*p* < 0.05, ^∗∗^*p* < 0.01). (b) Correlation between WNT16 and DCLK1 expression in undifferentiated cells and the degree of osteogenic differentiation as determined by matrix mineralization among T-MSCs from 10 different donors. (c) Representative images showing the degree of matrix mineralization and WNT16 and DCLK1 expression examined by RT-PCR.

Expression of WNT16, DPP4, DCLK1, IGFBP5, FBLN2, and HIGD1A was confirmed in other ND and OP clones as well as T-MSCs from four different donors using real-time quantitative PCR. We found that WNT16 and DCLK1 expression recapitulated the results of RNA sequencing analysis, with increased expression in OP clones. In MSCs, these genes were expressed in levels between ND and OP clones (Figure 4(a)), supporting the idea that a heterogeneous population of progenitors is present in MSCs. Expression levels of other genes were similar between groups.

Next, we questioned whether the expression levels of these genes could be used to predict the osteogenic differentiation potential of undifferentiated T-MSCs by examining the correlation between osteogenic differentiation and WNT16 and DCLK1 expression in undifferentiated cells from 10 different donors. Osteogenic differentiation was assessed by calcium deposition using Alizarin red S staining 3 weeks after differentiation. We found that WNT16 expression was positively correlated with the degree of osteoblast differentiation (Figure 4(b)). The expression of WNT16 prior to inducing differentiation was also correlated with the degree of osteoblast differentiation (Figure 4(c)). Furthermore, we examined the expression of WNT16 and DCLK1 during osteogenic differentiation of human BM-MSCs. We found that DCLK1 was highly expressed in predifferentiation stages and downregulated across all stages of osteoblast differentiation, whereas WNT16 expression was induced around day 7 postdifferentiation (Supplementary [Supplementary-material supplementary-material-1]).

### 3.5. The Correlation between WNT16 Expression and Osteogenic Capacity Is Specific for T-MSCs

**Figure 5:**
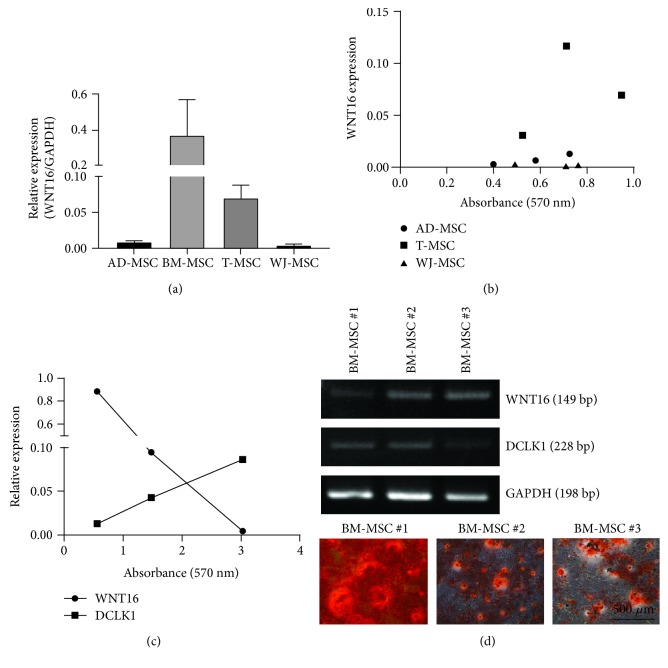
The correlation between WNT16 expression and osteogenic capacity is specific for T-MSCs. (a) WNT16 expression was examined in AD-, BM-, T-, and WJ-MSCs from three different donors using real-time quantitative PCR. Data are shown as mean ± SEM. (b) Matrix mineralization and WNT16 expression in three different donors of AD-, T-, and WJ-MSCs were presented. (c) Matrix mineralization and mRNA expression levels of WNT16 and DCLK1 in BM-MSCs were shown. (d) Images showing matrix mineralization and WNT16 and DCLK1 expression examined by RT-PCR.

Next, we expanded our observations to MSCs from different origins including BM, AD, and WJ. MSCs obtained from three different donors per each origin were subjected for mRNA extraction prior to inducing differentiation and examined for matrix mineralization 21 days postosteogenic differentiation. Expression levels of WNT16 in AD- or WJ-MSCs were near the detection limit (Figure 5(a)); thus, we could not draw a correlation between WNT16 expression and matrix mineralization in MSCs from those origins (Figure 5(b)). In BM-MSCs, interestingly, WNT16 expression showed a negative correlation with matrix mineralization while DCLK1 expression was positively correlated with osteogenic differentiation (Figures 5(c) and 5(d)). Overall, these data demonstrate a heterogeneity of MSCs among tissue origins, and the correlation between WNT16 expression and osteogenic capacity is specific for T-MSCs.

## 4. Discussion

Our results indicate that WNT16 expression predicts the osteogenic differentiation potential of T-MSCs. As we observed donor-to-donor variation in osteogenic differentiation of T-MSCs, we sought to identify a gene whose expression correlated with osteogenic potential. We performed single-cell cloning by limiting dilution and employed RNA sequencing to identify differentially expressed transcripts between OP and ND clones. We further analyzed the top five upregulated genes in OP clones and found that WNT16, the most highly upregulated gene in OP clones, was well correlated with the degree of osteogenic differentiation of T-MSCs.

Current methods widely used for isolating MSCs cannot adequately differentiate cells committed to a certain lineage. Attempts have been made to identify biomarkers that can classify a pure population committed to an osteogenic or adipogenic lineage [8]. However, sorted subpopulations often lose the self-renewal and multipotency properties of MSCs [21] and thus are not appropriate for use in stem cell therapy. Alternatively, the identification of predictable biomarkers that can distinguish purposes of use in specific contexts could enhance the clinical outcomes of stem cell therapy. Therefore, we examined monoclonal cells prone to differentiation into osteoblasts and identified WNT16 as a biomarker that could serve to select MSCs for stem cell therapy for bone-related diseases such as osteoporosis.

The WNT signaling pathway plays important roles in regulating bone homeostasis. Of the 19 WNT proteins identified in mammals, a relationship between WNT16 and cortical bone thickness was established by genome-wide association studies and is further supported by the characterization of WNT16-knockout mice [22]. Specifically, deletion of WNT16 reduces cortical bone thickness and femur strength, with a more severe phenotype in female than in male mice, suggesting that WNT16-based therapy could be effective for postmenopausal osteoporosis. This possibility is in agreement with a previous report demonstrating cortical bone thickening, but not trabecular bone thickening, after T-MSC injection in an ovariectomized mouse model of postmenopausal osteoporosis [17].

WNT16 signaling involves both canonical and noncanonical pathways that contribute to enhancements in bone strength via the inhibition of osteoclast differentiation [23]. Moverare-Skrtic et al. reported that WNT16 reduces osteoclastogenesis by inducing osteoprotegerin (OPG) expression which, in turn, interferes with the RANKL/RANK signaling pathway. This further highlights our previous finding that T-MSCs secrete OPG at much higher levels than AD- or BM-MSCs and inhibit RANKL-induced osteoclast activity [18]. These effects could be downstream of WNT16 signaling and may serve as one of the mechanisms of action of T-MSCs in the treatment of osteoporosis.

In addition, conditional deletion of WNT16 in early osteoblasts using RUNX2-creWNT16^flox/flox^ mice produces a phenotype similar to that after global deletion [23], demonstrating that WNT16 plays a critical and specific role in early stages of osteoblast differentiation. Our present finding demonstrated that T-MSCs with high WNT16 expression prior to inducing differentiation are readily differentiated into osteoblasts. This suggests that endogenous expression of WNT16 in preosteoblasts may prime cells to osteogenic lineage. However, further work is required to determine whether WNT16 expression is sufficient for the promotion of osteoblast differentiation using loss- or gain-of-function studies. In addition, mouse models of osteoporosis and/or bone fracture should be used to examine differences in the treatment efficacies of T-MSCs expressing high versus low levels of WNT16.

DCLK1 has been reported as a negative regulator of osteoblast differentiation via antagonizing RUNX2 activity and promoting microtubule polymerization [24, 25]. Under our observation, however, DCLK1 is highly expressed in BM-MSCs and then dramatically decreased after induction of osteogenic differentiation. In addition, data demonstrated that the higher the DCLK1 expression in BM-MSCs, the more cells differentiated into osteoblasts. Interestingly, DCLK1 and WNT16 mRNA levels showed an opposite pattern of expression. It would be interesting to investigate whether DCLK1 and WNT16 pathways are reciprocally regulated during osteogenesis of BM-MSCs.

## 5. Conclusions

In conclusion, we performed single-cell cloning and demonstrated heterogeneity of T-MSCs in terms of their osteogenic differentiation. We selected clones that maintained self-renewal properties and MSC surface marker expression but varied in osteogenic differentiation. Using RNA sequencing, we identified genes upregulated in OP clones and confirmed their expression in T-MSCs from different donors. We found that WNT16 expression in undifferentiated T-MSCs predicted osteogenic differentiation. The correlation between WNT16 expression and osteoblast differentiation was shown to be specific for T-MSCs. Our results suggest that the therapeutic application of T-MSCs expressing high levels of WNT16 could be efficacious in treating osteoporosis.

## Figures and Tables

**Table 1:** Sequences of primers used for real-time quantitative PCR.

Gene	GeneBank accession	Primer sequence	
		Forward (5′–3′)	Reverse (5′–3′)
GAPDH	NM_002046	CACATCGCTCAGACACCATG	TGACGGTGCCATGGAATTTG
WNT16	NM_016087	AGTATGGCATGTGGTTCAGCA	GCGGCAGTCTACTGACATCAA
DPP4	NM_001935	AGTGGCACGGCAACACATT	AGAGCTTCTATCCCGATGACTT
DCLK1	NM_001195415	ACTTCGACGAGCGGGATAAG	GGGCCTCAAAAGATCGGAACC
IGFBP5	NM_000599	TGACCGCAAAGGATTCTACAAG	CGTCAACGTACTCCATGCCT
FBLN2	NM_001004019	CAGGTGGCCTCTAACACCATC	CTGCTTGCAGGGTCCATTGT
HIGD1A	NM_001099668	AAGAGGCACCATTCGTACCC	ACCAACAGTCATTGCTCCTACA
RUNX2	NM_001015051	CCGCCTCAGTGATTTAGGGC	GGGTCTGTAATCTGACTCTGTCC

**Table 2:** Transcripts up- or downregulated in OP clones compared to ND clones (fc2 and0 raw.p).

Transcript_ID	Gene_Symbol	Description	OP/ND.fc
NM_016087	WNT16	Wnt family member 16	4.92
NM_001935	DPP4	Dipeptidyl peptidase 4	3.98
NM_001195415	DCLK1	Doublecortin-like kinase 1	3.90
NM_000599	IGFBP5	Insulin-like growth factor binding protein 5	2.78
NM_001004019	FBLN2	Fibulin 2	2.43
NM_001099668	HIGD1A	HIG1 hypoxia-inducible domain family member 1A	2.33
NM_014737	RASSF2	Ras association domain family member 2	2.07
NM_001322462	SH3BP5L	SH3 binding domain protein 5 like	-2.52
NM_001009939	SEPT5	Septin 5	-2.15

## Data Availability

The data used to support the findings of this study are available from the corresponding author upon request.

## Supplementary Materials

Supplementary Materials Supplementary Figure 1: transcriptome sequencing of three ND and three OP clones was performed, followed by further analysis of GO functional categories. Gene set enrichment of DEGs according to the GO categories of biological process, molecular function, and cellular component (^∗^*p* < 0.05, ^∗∗∗^*p* < 0.001). Supplementary Figure 2: time-course expression of (A) WNT16 and (B) DCLK1 in human BM-MSCs induced for osteogenic differentiation. Expression levels are normalized to those on differentiation day 0. Data are shown as mean ± SEM and were analyzed using one-way ANOVA. Different letters indicate significant differences between experimental groups (*n* = 3, *p* < 0.05).

## References

[1] Johnell O., Kanis J. A. (2006). An estimate of the worldwide prevalence and disability associated with osteoporotic fractures. *Osteoporosis International*.

[2] Phetfong J., Sanvoranart T., Nartprayut K. (2016). Osteoporosis: the current status of mesenchymal stem cell-based therapy. *Cellular & Molecular Biology Letters*.

[3] Petite H., Viateau V., Bensaïd W. (2000). Tissue-engineered bone regeneration. *Nature Biotechnology*.

[4] Horwitz E. M., Gordon P. L., Koo W. K. K. (2002). Isolated allogeneic bone marrow-derived mesenchymal cells engraft and stimulate growth in children with osteogenesis imperfecta: implications for cell therapy of bone. *Proceedings of the National Academy of Sciences of the United States of America*.

[5] Larsen K. H., Frederiksen C. M., Burns J. S., Abdallah B. M., Kassem M. (2010). Identifying a molecular phenotype for bone marrow stromal cells with in vivo bone-forming capacity. *Journal of Bone and Mineral Research*.

[6] Fukiage K., Aoyama T., Shibata K. R. (2008). Expression of vascular cell adhesion molecule-1 indicates the differentiation potential of human bone marrow stromal cells. *Biochemical and Biophysical Research Communications*.

[7] Kaltz N., Ringe J., Holzwarth C. (2010). Novel markers of mesenchymal stem cells defined by genome-wide gene expression analysis of stromal cells from different sources. *Experimental Cell Research*.

[8] Rostovskaya M., Anastassiadis K. (2012). Differential expression of surface markers in mouse bone marrow mesenchymal stromal cell subpopulations with distinct lineage commitment. *PLoS One*.

[9] Granéli C., Thorfve A., Ruetschi U. (2014). Novel markers of osteogenic and adipogenic differentiation of human bone marrow stromal cells identified using a quantitative proteomics approach. *Stem Cell Research*.

[10] Janjanin S., Djouad F., Shanti R. M. (2008). Human palatine tonsil: a new potential tissue source of multipotent mesenchymal progenitor cells. *Arthritis Research & Therapy*.

[11] Ryu K. H., Cho K. A., Park H. S. (2012). Tonsil-derived mesenchymal stromal cells: evaluation of biologic, immunologic and genetic factors for successful banking. *Cytotherapy*.

[12] Kim S. Y., Kim Y. R., Park W. J. (2015). Characterisation of insulin-producing cells differentiated from tonsil derived mesenchymal stem cells. *Differentiation*.

[13] Cho K. A., Park M., Kim Y. H., Woo S. Y., Ryu K. H. (2017). RNA sequencing reveals a transcriptomic portrait of human mesenchymal stem cells from bone marrow, adipose tissue, and palatine tonsils. *Scientific Reports*.

[14] Yu Y., Park Y. S., Kim H. S. (2014). Characterization of long-term in vitro culture-related alterations of human tonsil-derived mesenchymal stem cells: role for CCN1 in replicative senescence-associated increase in osteogenic differentiation. *Journal of Anatomy*.

[15] Kim Y. H., Park M., Cho K. A. (2016). Tonsil-derived mesenchymal stem cells promote bone mineralization and reduce marrow and visceral adiposity in a mouse model of senile osteoporosis. *Stem Cells and Development*.

[16] Kim G., Park Y. S., Lee Y. (2018). Tonsil-derived mesenchymal stem cell-embedded in situ crosslinkable gelatin hydrogel therapy recovers postmenopausal osteoporosis through bone regeneration. *PLoS One*.

[17] Kim G., Jin Y. M., Yu Y. (2018). Double intratibial injection of human tonsil-derived mesenchymal stromal cells recovers postmenopausal osteoporotic bone mass. *Cytotherapy*.

[18] Cho K. A., Park M., Kim Y. H., Ryu K. H., Woo S. Y. (2017). Mesenchymal stem cells inhibit RANK-RANKL interactions between osteoclasts and Th17 cells via osteoprotegerin activity. *Oncotarget*.

[19] Kim D., Langmead B., Salzberg S. L. (2015). HISAT: a fast spliced aligner with low memory requirements. *Nature Methods*.

[20] Pertea M., Kim D., Pertea G. M., Leek J. T., Salzberg S. L. (2016). Transcript-level expression analysis of RNA-seq experiments with HISAT, StringTie and Ballgown. *Nature Protocols*.

[21] Freeman B. T., Jung J. P., Ogle B. M. (2015). Single-cell RNA-Seq of bone marrow-derived mesenchymal stem cells reveals unique profiles of lineage priming. *PLoS One*.

[22] Zheng H. F., Tobias J. H., Duncan E. (2012). WNT16 influences bone mineral density, cortical bone thickness, bone strength, and osteoporotic fracture risk. *PLoS Genetics*.

[23] Movérare-Skrtic S., Henning P., Liu X. (2014). Osteoblast-derived WNT16 represses osteoclastogenesis and prevents cortical bone fragility fractures. *Nature Medicine*.

[24] Zhao M., Ko S. Y., Liu J. H. (2009). Inhibition of microtubule assembly in osteoblasts stimulates bone morphogenetic protein 2 expression and bone formation through transcription factor Gli2. *Molecular and Cellular Biology*.

[25] Zou W., Greenblatt M. B., Brady N. (2013). The microtubule-associated protein DCAMKL1 regulates osteoblast function via repression of Runx2. *The Journal of Experimental Medicine*.

