# Supplementary material for: Identification of WNT16 as a Predictable Biomarker for Accelerated Osteogenic Differentiation of Tonsil-Derived Mesenchymal Stem Cells In Vitro
Source: Stem Cells Int. 2019 Sep 10;2019:8503148. doi: 10.1155/2019/8503148 (PMC6754949; doi:10.1155/2019/8503148)

Identification of WNT16 as a predictable biomarker for osteogenic differentiation of tonsil-derived mesenchymal stem cells

Yu-Hee Kim[1], Kyung-Ah Cho[1], Hyun-Ji Lee[1], Minhwa Park[1], Han Su Kim[2], Joo-Won Park[3], So-Youn Woo[1], Kyung-Ha Ryu[4,*]

[1]Department of Microbiology, College of Medicine, Ewha Womans University, Seoul 07804, South Korea

[2]Department of Otolaryngology, College of Medicine, Ewha Womans University, Seoul 07985, South Korea

[3]Department of Biochemistry, College of Medicine, Ewha Womans University, Seoul 07804, South Korea

[4]Department of Pediatrics, College of Medicine, Ewha Womans University, Seoul 07804, South Korea

Correspondence: Kyung-Ha Ryu, M.D., Ph.D., Department of Pediatrics, College of Medicine, Ewha Womans University, Seoul 07804, South Korea. Tel: 82-2-6986-1666, E-mail: ykh@ewha.ac.kr

Supplementary Figure 1. Transcriptome sequencing of three ND and three OP clones was performed, followed by further analysis of GO functional categories. Gene set enrichment of DEGs according to the GO categories of biological process, molecular function, and cellular component (*$p<0.05$, ***$p<0.001$).

Supplementary Figure 2. Time-course expression of (A) WNT16 and (B) DCLK1 in human BM-MSCs induced for osteogenic differentiation. Expression levels are normalized to those on differentiation day 0. Data are shown as mean ± SEM and were analyzed using one-way ANOVA. Different letters indicate significant differences between experimental groups (n=3, $p<0.05$).

Supplementary Figure 1.

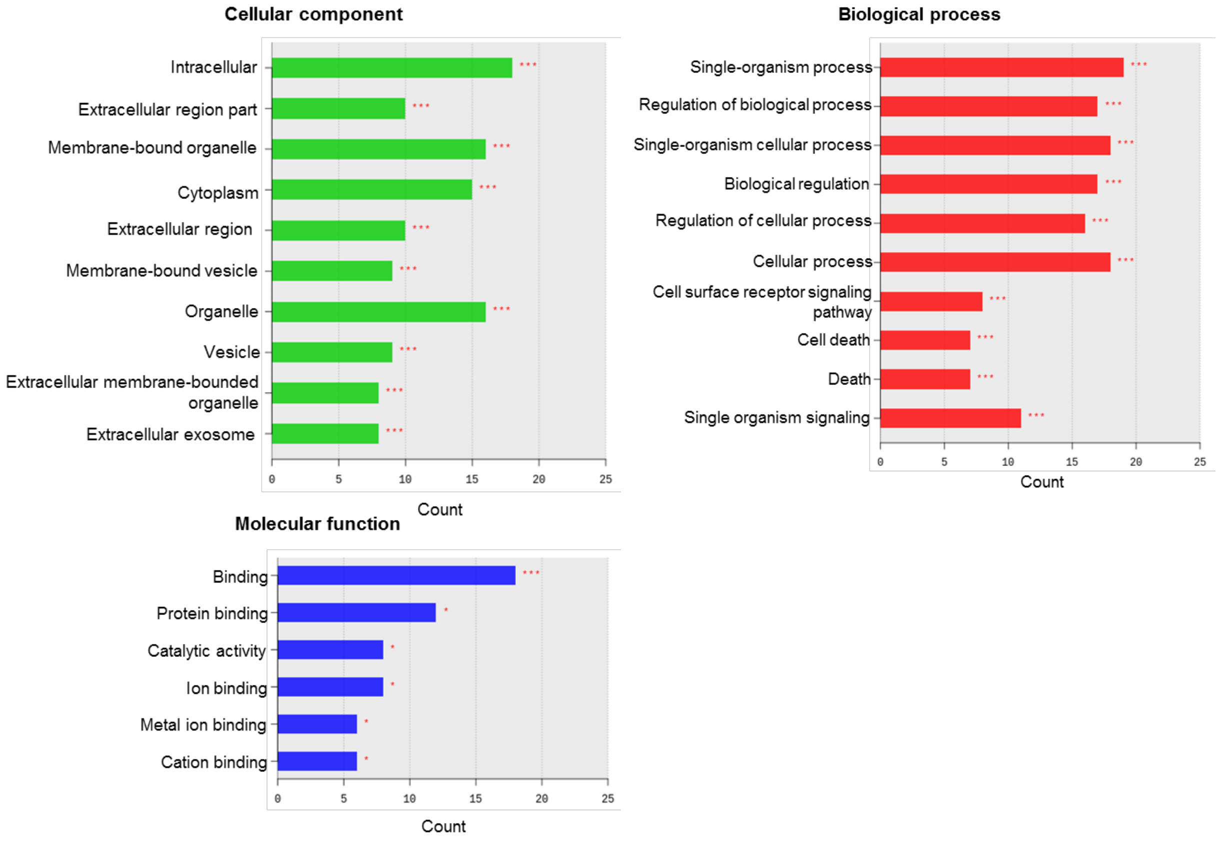

Supplementary Figure 2.

A

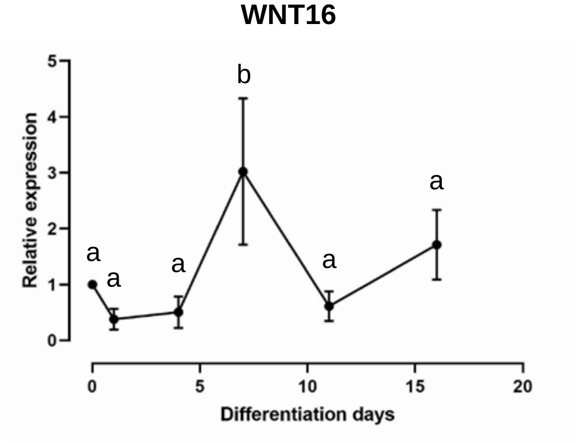

B

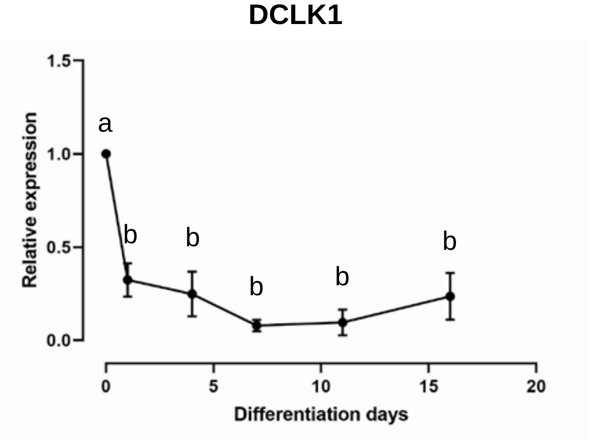

Supplement: Supplementary Materials — Supplementary Figure 1: transcriptome sequencing of three ND and three OP clones was performed, followed by further analysis of GO functional categories. Gene set enrichment of DEGs according to the GO categories of biological process, molecular function, and cellular component (∗p < 0.05, ∗∗∗p < 0.001). Supplementary Figure 2: time-course expression of (A) WNT16 and (B) DCLK1 in human BM-MSCs induced for osteogenic differentiation. Expression levels are normalized to those on differentiation day 0. Data are shown as mean ± SEM and were analyzed using one-way ANOVA. Different letters indicate significant differences between experimental groups (n = 3, p < 0.05). [file 8503148.f1.pdf]
